# Influence of parity and reproductive stage on the prevalence of *Mycoplasma hyopneumoniae* in breeding animals in belgian farrow-to-finish pig herds

**DOI:** 10.1186/s40813-022-00267-w

**Published:** 2022-06-09

**Authors:** Evelien Biebaut, Ilias Chantziaras, Filip Boyen, Bert Devriendt, Freddy Haesebrouck, Charles-Oliver Gomez-Duran, Dominiek Maes

**Affiliations:** 1 Department of Internal Medicine, Reproduction and Population Medicine, Faculty of Veterinary Medicine, Ghent University, Merelbeke, Belgium; 2 Department of Pathobiology, Pharmacology and Zoological Medicine, Faculty of Veterinary Medicine, Ghent University, Merelbeke, Belgium; 3 Department of Translational Physiology, Infectiology and Public Health, Faculty of Veterinary Medicine, Ghent University, Merelbeke, Belgium; 4 Boehringer Ingelheim Vetmedica GmbH, Ingelheim, Germany

**Keywords:** *Mycoplasma hyopneumoniae*, Breeding animals, Prevalence

## Abstract

**Background:**

Dam-to-piglet transmission plays an important role in the epidemiology of enzootic pneumonia on farms. Although *Mycoplasma hyopneumoniae* (*M. hyopneumoniae*) infections in breeding animals are often subclinical, their control could have a positive effect on *M. hyopneumoniae* infection levels in fattening pigs. This study investigated the presence of *M. hyopneumoniae* in the breeding population of ten Belgian farrow-to-finish farms suspected by the herd veterinarian to be *M. hyopneumoniae* infected. Gilt vaccination against *M. hyopneumoniae* prior to first insemination was practiced on nine of the ten farms. At four different time points in the reproductive cycle 20 animals were sampled on each farm, namely 30–40 days of gestation, 75–85 days of gestation, 3–5 days after farrowing, and 1–3 days after weaning. In total, tracheobronchial swabs and blood samples were collected from 344 gilts and 456 sows (n = 80/farm). Swabs were analysed for the presence of *M. hyopneumoniae* DNA using nested PCR and *M. hyopneumoniae*-specific antibodies were detected in serum with a commercial ELISA. Generalized linear mixed models with farm as random factor were used to test the effect of time point in the reproductive cycle and parity on *M. hyopneumoniae* PCR prevalence and seroprevalence.

**Results:**

*M. hyopneumoniae* PCR prevalence ranged between 0% and 43.8% at the farm level and the seroprevalence between 32.5% and 93.8%. Gilts were significantly more *M. hyopneumoniae* PCR positive than sows at the 2-4th parity (P = 0.02) and > 4th parity (P = 0.02). At 30–40 days of gestation, significantly more breeding animals were PCR positive as compared to 75–85 days of gestation (P = 0.04), 3–5 days after farrowing (P = 0.02) and 1–3 days after weaning (P = 0.02). Gilts had significantly more often *M. hyopneumoniae*-specific antibodies than sows (P = 0.03).

**Conclusions:**

*M. hyopneumoniae* PCR prevalence varied a lot between farms and due to gilt vaccination the number of animals with *M. hyopneumoniae*-specific antibodies was high on most farms. Gilts were more often *M. hyopneumoniae* PCR positive than sows and positive animals were mostly found at 30–40 days of gestation. This emphasizes the importance of a sufficiently long quarantine period and proper gilt acclimation practices before introducing gilts to the sow herd.

**Supplementary Information:**

The online version contains supplementary material available at 10.1186/s40813-022-00267-w.

## Background

*Mycoplasma hyopneumoniae* (*M. hyopneumoniae*) is the primary agent of enzootic pneumonia (EP) in pigs causing significant economic losses in swine production worldwide [1, 2]. These losses are mainly present at the level of the grow-finishing pigs, but breeding gilts and sows are an important source of *M. hyopneumoniae* on the farm. Infections in breeding animals are mostly asymptomatic, but these animals can transmit the pathogen to their offspring in the farrowing unit [3–5]. The occurrence of *M. hyopneumoniae* in piglets at weaning is associated with the presence of EP-like lung lesions and the percentage of affected lungs at the moment of slaughter [6, 7]. The purchase of more than 120 gilts each year, the circulation of respiratory pathogens in the breeding population, and the presence of *M. hyopneumoniae* positive sows in the farrowing unit are risk factors for a higher *M. hyopneumoniae* prevalence at weaning [5, 8, 9]. If the presence of the pathogen in the breeding population can be reduced less piglets will be *M. hyopneumoniae* positive at weaning and the losses at the level of the grow-finishing pigs could decrease. In order to optimize *M. hyopneumoniae-*specific control measures in the breeding population (vaccination, acclimation), it is necessary to gain a better insight in the prevalence and epidemiology of *M. hyopneumoniae* in breeding animals.

Although most herds are endemically infected with *M. hyopneumoniae*, the presence of the pathogen and *M. hyopneumoniae*-specific antibodies in breeding animals may differ a lot between farms [5, 10, 11]. Several studies have focused on the link between sow parity and the presence of *M. hyopneumoniae* in sows. Most studies reported a higher PCR prevalence or seroprevalence of *M. hyopneumoniae* in gilts and/or young sows [3, 10, 12, 13], while others did not find such a correlation [14]. Besides the parity also the time point (TP) in the reproductive cycle may influence the presence of *M. hyopneumoniae*, especially in gilts [15]. Weaning of the piglets, moving the sows to the insemination unit, and later on during gestation to the group housing system are stressful periods for breeding animals [16, 17]. Stress can enhance but also suppress the immune system [18] influencing the susceptibility for infections and shedding of pathogens [19]. However, in a longitudinal study by Fablet et al. [11] a significant influence of TP on *M. hyopneumoniae* PCR prevalence could not be demonstrated. Previous studies addressing *M. hyopneumoniae* PCR prevalence or seroprevalence in breeding animals are mostly older studies including only one or a few herds. Furthermore, gestating sows were not moved to a group housing system and often only blood samples (seroprevalence) or swabs from the upper respiratory tract were taken. The chance of detecting *M. hyopneumoniae* is higher when samples are taken from the lower respiratory tract [20].

Since 2013, group housing of breeding animals between four weeks of gestation and one week before farrowing is obligatory in the European Union (Directive 2008/120/EC) [21]. However, little is known about the impact of this type of housing on the occurrence of infectious diseases in breeding animals. Therefore, it might be useful to investigate whether breeding animals are more often *M. hyopneumoniae* PCR positive at specific TPs in the reproductive cycle under group housing conditions.

The present study aimed to investigate the *M. hyopneumoniae* seroprevalence and infection status in 800 breeding animals from ten different herds in Belgium. The specific objectives were (1) to investigate the influence of parity on *M. hyopneumoniae* PCR prevalence and seroprevalence, (2) to investigate the influence of the TP in the reproductive cycle on *M. hyopneumoniae* PCR prevalence and seroprevalence and (3) to investigate the potential correlation between the infection status and seroprevalence.

## Materials and methods

### Study population

The study was performed after approval by the Ethical Committee for Animal Experiments of the Faculty of Veterinary Medicine and the Faculty of Bioscience Engineering, Ghent University (approval number EC2020-031). Ten Belgian farrow-to-finish farms were included in the study. The first five farms were sampled in 2020 between January and March and the other five farms in 2021 in the same months. Herd inclusion criteria were: no vaccination of sows against *M. hyopneumoniae*, at least part of the piglets raised at the same site, and willingness to participate. Farmers were allowed to practice vaccination of gilts against *M. hyopneumoniae* and both breeding of their own gilts or purchase of gilts was permitted. Farms were selected when the herd veterinarian suspected *M. hyopneumoniae* circulation in young piglets and/or the sow population based on historical information (serology/presence of the pathogen/coughing problems).

### Animals and sampling

On each farm a cross-sectional sampling was performed by sampling 80 breeding animals equally divided over four different TPs in the reproductive cycle; 30–40 (TP1) and 75–85 (TP2) days of gestation, 3–5 days after farrowing (TP3) and 1–3 days after weaning (TP4). On farms with a one- or three-week-production system all samples were taken on the same day, while two sampling moments were needed with approximately nine days in between for farms working in a four-week-production system. Ten clusters (farms) were needed for both the groups of sows and gilts to achieve a power of 80% to detect a difference in proportion of 0.05 between the two groups. In each cluster for the four selected TPs ten gilts and ten sows had to be sampled, meaning 800 pigs in total. With a cluster auto-correlation of 80% and 120% the power ranged from 0.70 to 0.88, respectively, which both were deemed satisfactory. If a farmer did not have ten gilts in a specific batch, all gilts were sampled and completed with sows until 20 animals were sampled at each TP. In each batch the 10 sampling sows were chosen randomly. In total, 800 breeding animals were sampled of which 344 were gilts and 456 were sows. In the group of the gilts 183 animals had not farrowed yet (TP1 and TP2) and 161 animals had farrowed once (TP3 and TP4). There were 285 2-4th parity animals and 171 > 4th parity animals in the group of the sows. From each animal blood and tracheobronchial swabs (TBS) were collected.

### Laboratory analysis

#### *Mycoplasma hyopneumoniae*-specific antibodies

Blood was collected in a sterile serum tube (clotted blood) by puncture of the jugular vein or *vena cava cranialis*. Samples were centrifuged at 1000*xg* and serum was stored at −20 °C until further analysis. To detect the presence of *M. hyopneumoniae*-specific antibodies, a commercial indirect ELISA (*M. hyo* Ab test, IDEXX Laboratories Inc., Westbrook, ME, USA) was used following the manufacturer’s instructions. Samples were considered positive if the sample to positive (S/P) ratio was higher than 0.40 and negative if the S/P ratio was equal to or lower than 0.40.

#### Nested PCR for *Mycoplasma hyopneumoniae* DNA detection

A sterile swab of 60 centimeters (sucking-catheter, Medinorm GmbH, Spiesen-Elversberg, Germany) was used for the tracheobronchial sampling [22]. All TBS samples were stored at −80 °C until further analysis. To test for the presence of *M. hyopneumoniae*, DNA was extracted from the TBS using a commercial kit (DNaesy® Blood & Tissue kit, Qiagen, Venlo, The Netherlands) and a nested PCR was performed according to the protocol described by Stärk et al. [23].

### Data analysis

All statistical analyses were performed using IBM SPSS version 27® (Armonk, New York, USA). Descriptive information (average, mean, minimum, maximum) regarding the various parameters included in this study was calculated. Test result (either PCR or ELISA) was selected as the dependent variable. We used generalized linear mixed models (GLMM) and fitted binomial logistic models with farm included as random factor and TP included as fixed factor. Two models were developed for the *M. hyopneumoniae* infection status (PCR). In the first one, the parameter ‘parity’ was used as binary (gilt or sow) and in the second one as categorical (gilt, 2-4th parity, > 4th parity). Furthermore, to process the serological data (ELISA), also two models were used including the same parameters as the infection status models. Categorical or binary fixed variables with absence of variability among their categories, meaning that more than 90% of the total samples belonged to a given category, were excluded for further statistical analysis. To correlate PCR and serology results the positive predictive value (PPV) and negative predictive value (NPV) were calculated considering the PCR and ELISA data as binary. When calculating the PPV_ELISA_ and NPV_ELISA_ the PCR test was considered as gold standard and vice versa. Moreover, a point-biserial correlation was performed to correlate PCR and serology results. Apart from that, a GLMM was used with PCR outcome as dependent, farm as random, TP as fixed variable and the precise optical density (OD) values as fixed variable to investigate the association between PCR and serology results. The higher the OD value, the higher the level of *M. hyopneumoniae*-specific antibodies in the serum. For all GLMM pairwise comparisons were run post-hoc for all the fixed factors and a sequential Sidak correction was applied to correct for multiple testing.

To see if differences in housing and management of the breeding animals had an influence on the presence of *M. hyopneumoniae* on a farm, the ten farms were split in five farms with the highest *M. hyopneumoniae* prevalence (HPF) and five farms with the lowest *M. hyopneumoniae* prevalence (LPF) after analyzing the PCR data.

## Results

### Herd characteristics

An overview of the herd characteristics is shown in Table 1. The median (min.-max.) number of sows in the ten herds was 440 (270–2400), and the average (min.-max.) parity number 3.7 (3.0–4.9). Three farms worked in a one-week production system, two farms in a three-week production system, and five farms in a four-week production system. Most farms (7/10) purchased gilts and they all respected a quarantine period of at least three weeks before introducing them to the sow herd. On all seven farms the quarantine unit was located in a separate stable. In two of the seven herds purchasing gilts the animals originated from an *M. hyopneumoniae* negative farm. Purchased gilts were vaccinated against *M. hyopneumoniae* only in the quarantine unit on four farms, on two farms the gilts were vaccinated at the supplier and in the quarantine unit, and on one farm *M. hyopneumoniae* vaccination was only done at the supplier. At two out of three farms rearing their own gilts, the gilts were vaccinated against *M. hyopneumoniae* during rearing and one farm (farm 9) did not practice *M. hyopneumoniae* vaccination. None of the farms vaccinated the gilts after moving them to the insemination unit. Similarly, none of the farms vaccinated the sows against *M. hyopneumoniae.* On all farms gilts had contact with the sows for the first time in the insemination unit. Mostly, gestating sows were brought in group housing around four weeks of gestation (8/10), only one farm waited until five weeks. On another farm, gestating animals were housed in group already from three days after insemination onwards. According to the farmer and/or the herd veterinarian there were some coughing problems in the breeding animals and/or in young piglets on six farms.

**Table 1:** Herd characteristics and PCR prevalence and seroprevalence of the ten farrow-to-finish farms included in the study

	Farm 1	**Farm 2**	Farm 3	Farm 4	**Farm 5**	**Farm 6**	**Farm 7**	**Farm 8**	Farm 9	Farm 10
Number of sows	450	**960**	430	270	**370**	**2400**	**1600**	**400**	280	1000
Breed	Topigs20	**Danbred**	Danbred	Danbred	**Topigs20 - TN70**	**Topigs20**	**Danbred**	**Danbred**	Hypor	TN70
Batch farrowing system for the sows (…week system)	4	**4**	4	4	**3**	**1**	**1**	**4**	3	1
Average parity number	3.2	**4.0**	3.7	4.9	**3.0**	**3.7**	**3.6**	**4.0**	3.8	3.0
Purchase of gilts	+	**−**	+	+	**+**	**+**	**+**	**−**	−	+
Quarantine for purchased gilts	+	**N.A**	+	+	**+**	**+**	**+**	**N.A**	N.A	+
Gilts purchased from *Mhyo* negative farm	−	**N.A**	+	+	**−**	**−**	**−**	**N.A**	N.A	−
Duration of quarantine (weeks)	4	**N.A**	7	4	**6**	**3**	**6**	**N.A**	N.A	5
Quarantine unit located in separate stable	+	**N.A**	+	+	**+**	**+**	**+**	**N.A**	N.A	+
*Mhyo* vaccination of gilts										
At the supplier	+				**+**					+
In the quarantine			+	+	**+**	**+**	**+**			+
During rearing		**+**						**+**		
*Mhyo* vaccination of sows	−	**−**	−	−	**−**	**−**	**−**	**−**	−	−
First contact gilts-sows in insemination unit	+	**+**	+	+	**+**	**+**	**+**	**+**	+	+
Group housing after … days of gestation	28	**28**	25	25	**28**	**3**	**28**	**28**	35	28
Clinical signs of respiratory disorders										
Sows/gilts	−	**+**	−	−	**−**	**+**	**+**	**+**	+	−
Young piglets	−	**+**	−	−	**+**	**−**	**−**	**−**	+	−
*Mhyo* positive TBS (%, number)	2.5 2/80	**43.8** **35/80**	3.8 3/80	0.0 0/80	**33.8** **27/80**	**20.0** **16/80**	**18.8** **15/80**	**40.0** **32/80**	5.0 4/80	7.5 6/80
*Mhyo* seropositive animals (%, number)	66.3 53/80	**92.5** **74/80**	76.3 61/80	73.8 59/80	**87.5** **70/80**	**91.3** **73/80**	**85.0** **68/80**	**93.8** **75/80**	32.5 26/80	95.0 76/80

*Mhyo*: *Mycoplasma hyopneumoniae*; TBS: tracheobronchial swab; N.A.: not applicable; +: yes; −: no; Bold = five farms with the highest *M. hyopneumoniae* prevalence (≥ 18.8%)

### PCR testing for *Mycoplasma hyopneumoniae* prevalence

**Fig. 1:**
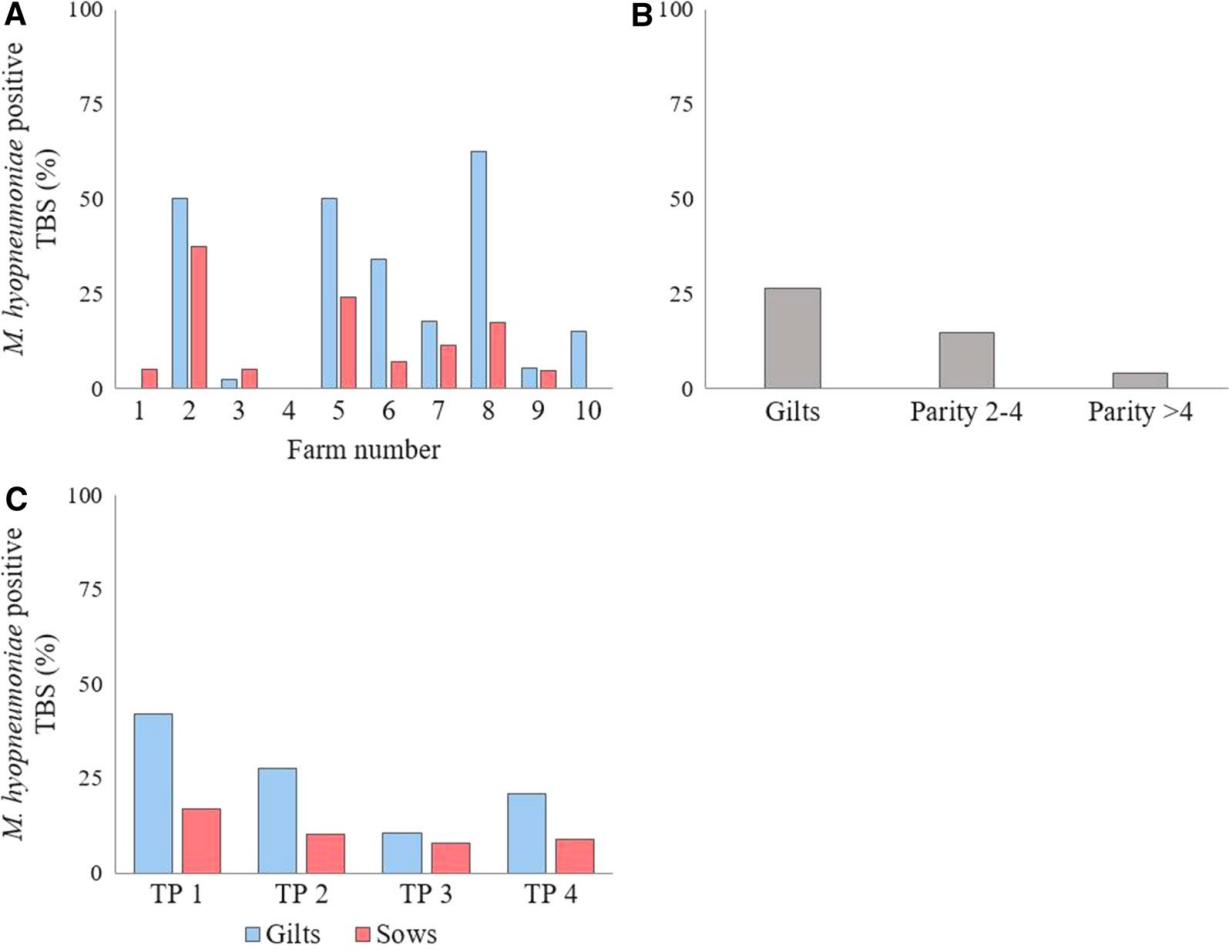
*M. hyopneumoniae* PCR prevalence on ten Belgian farrow-to-finish farms. On ten Belgian farrow-to-finish farms tracheobronchial swabs (TBS) were taken of 80 breeding animals (n = 800) at four different time points (TPs) in the reproductive cycle (20 animals at each TP); 30–40 days (TP1) and 75–85 days (TP2) of gestation, 3–5 days after farrowing (TP3) and 1–3 days after weaning (TP4). TBS were analyzed with nested PCR for the presence of *M. hyopneumoniae* DNA. The percentage of *M. hyopneumoniae* positive TBS is shown **A** for gilts and sows on each farm, **B** for the different parity groups, and **C** at the different TPs

The *M. hyopneumoniae* prevalence ranged between 0% and 43.8% at the farm level. The ten farms could be split in five LPF with *M. hyopneumoniae* prevalence ≤ 7.5% and five other HPF with *M. hyopneumoniae* prevalence ≥ 18.8% and ≤ 43.8% (Table 1). On the LPF the median number of sows was 430 and on the HPF 960. Overall, 26.5% of the gilts (91/344) and 10.7% of the sows (49/456) were *M. hyopneumoniae* positive. At the farm level the prevalence ranged between 0-62.5% and 0–37.5% for gilts and sows, respectively (Fig. 1A). The *M. hyopneumoniae* prevalence was the highest in the gilts and decreased over the parity groups (Fig. 1B). For the breeding animals in total, 29.5% (59/200), 17.5% (35/200), 9.0% (18/200), and 14.0% (28/200) were *M. hyopneumoniae* positive at TP1, TP2, TP3 and TP4, respectively. The percentages of *M. hyopneumoniae* positive gilts and sows at the different TPs are shown in Fig. 1C.

According to the statistical model, gilts were significantly more *M. hyopneumoniae* positive than animals with parity 2–4 (P = 0.02) and parity > 4 (P = 0.02). Animals with parity 2–4 tended to be more often *M. hyopneumoniae* positive than animals with parity > 4, although this difference was not statistically significant (P = 0.11). Pairwise comparisons of the different TPs showed that breeding animals in general were significantly more *M. hyopneumoniae* positive at TP1 compared to TP2 (P = 0.04), TP3 (P = 0.02) and TP4 (P = 0.02). Within the group of gilts, the same significant results were observed when TP1 was compared to TP2 (P = 0.04), TP3 (P = 0.02) and TP4 (P = 0.02). Pairwise comparisons between the other TPs were not statistically significant. For the group of sows, there were no statistically significant differences between the TPs. Detailed results of the different statistical models are provided as supplementary data (Additional file 1).

### ELISA testing for *Mycoplasma hyopneumoniae* seroprevalence

**Fig. 2:**
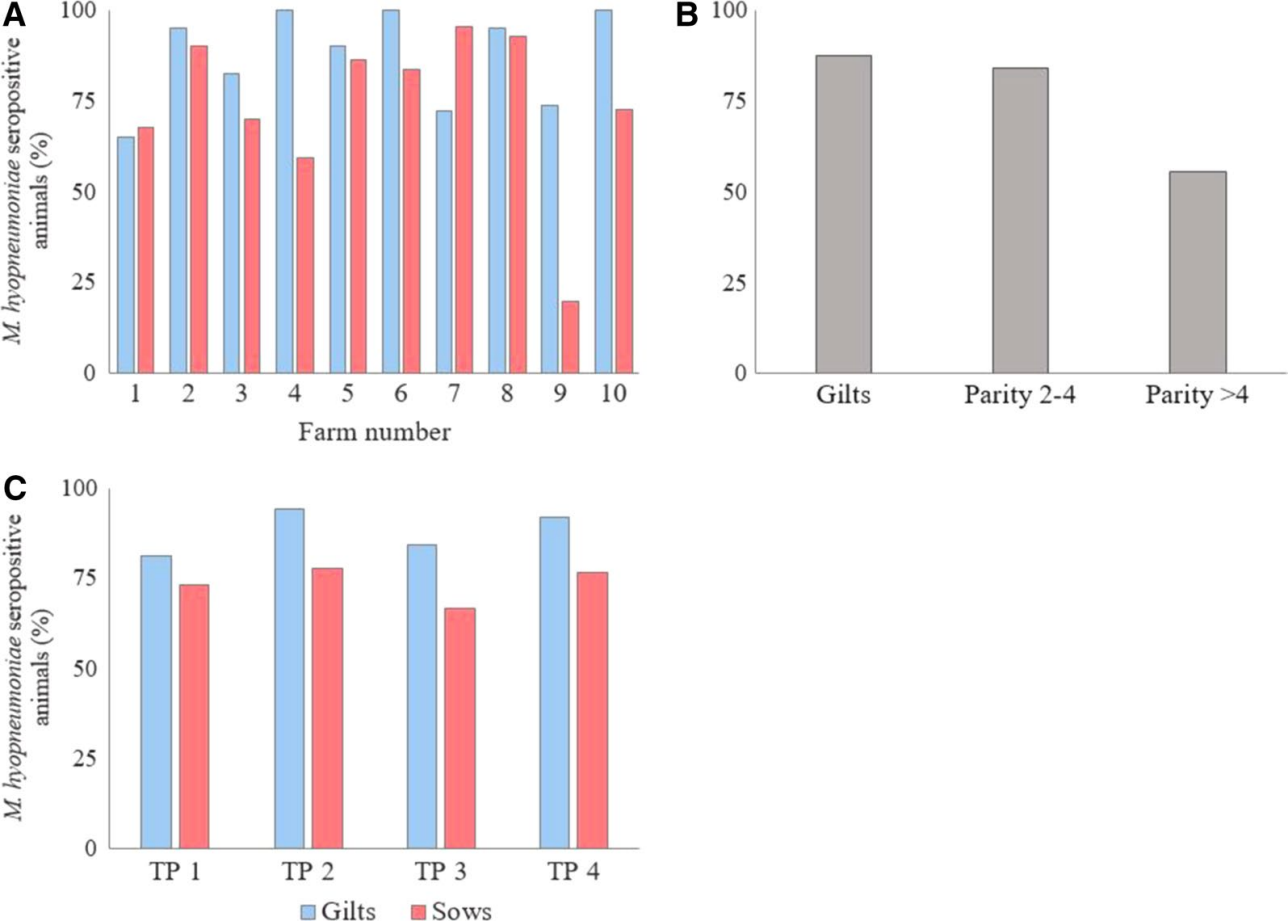
*M. hyopneumoniae* seroprevalence on ten Belgian farrow-to-finish farms. On ten Belgian farrow-to-finish farms blood was taken from 80 breeding animals (n = 800) at four different time points (TPs) in the reproductive cycle (20 animals at each TP); 30–40 days (TP1) and 75–85 days (TP2) of gestation, 3–5 days after farrowing (TP3) and 1–3 days after weaning (TP4). On all farms, except farm 9, gilts were vaccinated against *M. hyopneumoniae*. Serum was analyzed with a commercial ELISA for the presence of *M. hyopneumoniae*-specific antibodies. The percentage of *M. hyopneumoniae* seropositive animals is shown **A** for gilts and sows on each farm, **B** for the different parity groups, and **C** at the different TPs

The seroprevalence for *M. hyopneumoniae* ranged between 32.5% and 93.8% at the farm level. Farm 9 had by far the lowest seroprevalence (32.5%), while the seroprevalence for the other farms was higher than 66% (Table 1). Overall, 87.5% of the gilts (301/344) and 73.2% of the sows (334/456) had *M. hyopneumoniae*-specific antibodies. At the farm level the seroprevalence ranged between 65.0-100% and 19.7–95.5% for gilts and sows, respectively (Fig. 2A). Less sows with parity > 4 had *M. hyopneumoniae*-specific antibodies compared to the other parity groups (Fig. 2B). For the breeding animals in total, 77.0% (154/200), 84.5% (169/200), 73.0% (146/200), and 83.0% (166/200) had *M. hyopneumoniae*-specific antibodies at TP1, TP2, TP3 and TP4, respectively. The percentages of gilts and sows with *M. hyopneumoniae*-specific antibodies at the different TPs are shown in Fig. 2C.

According to the statistical model, significantly more gilts had *M. hyopneumoniae*-specific antibodies compared to sows (P = 0.03), and significantly less sows with parity > 4 were seropositive compared to sows with parity 2–4 (P = 0.02) and gilts (P = 0.02). Pairwise comparisons showed no statistically significant differences for the seroprevalence in breeding gilts or sows at the different TPs. Detailed results of the different statistical models are provided as supplementary data (Additional file 2).

### Correlation between PCR and ELISA

To investigate whether the PCR prevalence of *M. hyopneumoniae* on a farm was correlated with the ELISA prevalence, the PPV and NPV were calculated and a point-biserial correlation and GLMM were used. The PPV_PCR_ was 90.7% (127/140) and the NPV_PCR_ was 23.0% (152/660). The PPV_ELISA_ was 20.0% (127/635) and the NPV_ELISA_ was 92.1% (152/165). An overview of the test results is shown in Table 2. A low (R^2^=0.27) but statistically significant (P < 0.01) correlation was observed between the OD-values of the ELISA and a PCR positive result. *M. hyopneumoniae* PCR positive gilts had more often a high OD-value compared to *M. hyopneumoniae* PCR positive sows (P = 0.03). Considering that the farms had identical random effects and holding TP4 fixed, the odds for *M. hyopneumoniae* PCR positive gilts to have a high OD-value was 5.5 (P = 0.001), while the odds for PCR positive sows to have a high OD-value was 0.36 (P < 0.001).

**Table 2:** Test characteristics for ELISA and PCR

		PCR		Total
		Positive	Negative	
ELISA	Positive	127	508	635
	Negative	13	152	165
	Total	140	660	800
PPV_ELISA_	20.0%		PPV_PCR_	90.7%
NPV_ELISA_	92.1%		NPV_PCR_	23.0%

On ten Belgian farrow-to-finish farms 80 (n = 800) tracheobronchial swabs and blood samples were collected from breeding animals. Swabs were tested for the presence of *M. hyopneumoniae* DNA with nested PCR and blood was analyzed for the presence of *M. hyopneumoniae*-specific antibodies with a commercial ELISA. The positive predictive value (PPV) and negative predictive value (NPV) were calculated for both tests. PCR was set as gold standard when the test characteristics for the ELISA were calculated, and vice versa

## Discussion

Ten Belgian farrow-to-finish farms were visited in this study and all samples were taken between January and March in either 2020 or 2021. Only farms where the veterinarian suspected that there was *M. hyopneumoniae* circulation were included. Consequently, the results might not be extrapolated to the entire Belgian population of farrow-to-finish farms as only suspected EP-problem farms were included. Furthermore, samples were taken in winter period to have a higher chance of finding *M. hyopneumoniae* positive animals. When pigs are raised during cold, rainy periods, the chance on being infected with *M. hyopneumoniae* is higher [24, 25].

Based on PCR prevalence the farms could be divided in five HPF (≥ 18.8%) and five LPF (≤ 7.5%). On all the HPF and one LPF coughing problems were observed by the farmer and/or the herd veterinarian in the breeding animals and/or in young piglets. Unfortunately, coughing was not observed nor quantified during the samplings and the presence of other respiratory pathogens in the breeding population was not investigated. Therefore, coughing problems might be associated with *M. hyopneumoniae* PCR prevalence, but this could not be confirmed. The HPF were larger and they had more breeding animals. Animals on large farms, purchasing a larger number of gilts each year, have a higher risk of being infected with *M. hyopneumoniae* [9, 26]. For the other herd characteristics there were no clear differences between high and low prevalence farms. Gilts were purchased on four LPF and on three HPF. On two LPF, the gilts originated from an *M. hyopneumoniae* free farm. The duration of the quarantine period might be important but lasted on average five weeks in both groups [27]. Furthermore, previous research showed an influence of the batch farrowing system on *M. hyopneumoniae* PCR prevalence or seroprevalence in sows and suckling pigs [9, 10], whereas in our study it seemed to have no influence. Vaccination of gilts against *M. hyopneumoniae* was practiced on all farms except one. It is recommended to vaccinate gilts against *M. hyopneumoniae* to reduce shedding and transmission, but vaccination will not prevent infection [15, 28–30]. This is supported by our data, as despite gilt vaccination the number of *M. hyopneumoniae* infected animals on a farm can be high.

Between the farms the variation of *M. hyopneumoniae* PCR prevalence was high. Although *M. hyopneumoniae* circulation was suspected, the PCR prevalence was low or even zero in the breeding animals on some farms [13]. For the seroprevalence on the other hand, the percentages were high and there was less variation between farms. A high seroprevalence in gilts was expected as on nine out of ten farms gilts were vaccinated against *M. hyopneumoniae*. Due to gilt vaccination, it is not surprising that the number of breeding animals with *M. hyopneumoniae*-specific antibodies decreased with increasing parity. The concentration of antibodies in blood will decrease over time in the absence of a natural infection boosting the antibody response. On the farm not practicing gilt vaccination (farm 9), the seroprevalence in gilts was 73.9%, meaning that *M. hyopneumoniae* infections were present in the gilt population and the antibodies in these animals were due to natural infections. On the other farms, it is not known whether serum antibodies were due to vaccination, infection, or both. This highlights the need to develop DIVA compliant vaccines against *M. hyopneumoniae* [31]. In this study, gilts were significantly more often infected with *M. hyopneumoniae*, and gilts had more often *M. hyopneumoniae*-specific antibodies compared to sows, which is in line with previous findings [3, 10, 12, 13]. However, in most studies *M. hyopneumoniae* PCR prevalence or seroprevalence was investigated in breeding animals only to draw conclusions on their offspring [5, 32]. Only a few studies investigated the PCR prevalence or seroprevalence in breeding animals in a larger number of herds [11, 13]. Furthermore, swabs for the detection of *M. hyopneumoniae* DNA were mostly taken in the upper respiratory tract using PCR analysis while the sensitivity of TBS is higher [20]. In the present study, breeding animals on ten different farms were included and TBS were taken. No samples were taken from the offspring of those breeding animals. Moreover, group housing of gestating sows is only obliged in the EU since 2013. This implies that results from studies conducted in the EU before 2013 or outside the EU, where pregnant sows are housed in individual crates, might not be applicable to farms with group housing systems. In a German and a French study, including 67 and 5 herds respectively, all herds had *M. hyopneumoniae* seropositive sows and the TP in the reproductive cycle had no influence on the seroprevalence, which is in line with our findings [10, 11]. However, comparing seroprevalences between studies is difficult because results depend on the *M. hyopneumoniae* vaccination status of the breeding population. In their study, Fablet et al. [11] took swabs from the upper respiratory tract from breeding animals in a longitudinal study design. Similar to our study they did not find *M. hyopneumoniae* on every farm (4/5) and breeding animals were more often PCR positive at 9 weeks before farrowing compared to later TPs in the reproductive cycle, but this result was not significant [11]. In a Brazilian study in which gilts of an *M. hyopneumoniae* positive farm were followed from birth till weaning of their first litter, the highest *M. hyopneumoniae* PCR prevalence was seen shortly before first insemination [15]. In line with our findings, gilts were more often *M. hyopneumoniae* PCR positive in the first half of gestation compared to the remainder of their reproductive cycle [15]. However, both studies [11, 15] were longitudinal studies making it difficult to fully compare with the results of our cross-sectional study.

On all ten farms gilts were introduced to the sow herd in the insemination unit and gestating sows were housed in group from approximately four weeks of gestation onwards on eight farms. *M. hyopneumoniae* PCR prevalence was the highest at 30–40 days of gestation in gilts (significant) as well as in sows (not significant). This supports the theory that merging gilts and sows increases the risk for being *M. hyopneumoniae* PCR positive. Since no samples were taken in our study from the gilts in the quarantine stable or before insemination, it is not possible to indicate if *M. hyopneumoniae* positive sows infected the new gilts or vice versa. However, before introducing gilts to the sow population good acclimation of the gilts is necessary [33]. A sufficiently long quarantine period is recommended to allow proper vaccination, to perform diagnostic tests and to cover the incubation period of the most important pathogens. Furthermore, gilts should be immunized against most important pathogens before mixing them with sows [33]. When gilts are purchased from an *M. hyopneumoniae* negative source into an endemically infected farm, good immunization is even more important [34]. Vaccination against *M. hyopneumoniae* is the most commonly applied immunization, as other methods like contact with culled sows are less controlled [26, 33].

Serological testing for the presence of *M. hyopneumoniae*-specific antibodies is commonly practiced to perform herd-level monitoring. Nevertheless, the use of serology has its limitations to assess *M. hyopneumoniae* epidemiology. First, seroconversion after a natural *M. hyopneumoniae* infection is variable making it difficult to detect an infection in the early-stage [35–37]. Second, vaccination against *M. hyopneumoniae* is frequently practiced and ELISA tests cannot distinguish antibodies induced by vaccination or by natural infection [38]. If an animal was *M. hyopneumoniae* seronegative, it was also *M. hyopneumoniae* PCR negative in 92.1% of the cases (NPV_ELISA_). Animals being seronegative but PCR positive might have been in the early stages of infection [35, 36]. More important is that only 20% of the seropositive animals (PPV_ELISA_) were positive for *M. hyopneumoniae* on PCR. The outcome of the test characteristics for both PCR and ELISA were obviously influenced by the fact that on nine farms gilts were vaccinated against *M. hyopneumoniae.* However, the results of the PPV_ELISA_ and NPV_ELISA_ are of value for the field as *M. hyopneumoniae* vaccination of gilts is commonly practiced. Most gilts will have *M. hyopneumoniae*-specific antibodies even if the pathogen is not circulating on the farm. This demonstrates that to estimate the *M. hyopneumoniae* prevalence in breeding animals on farms endemically infected with the pathogen and practicing gilt vaccination, it is necessary to take swabs and perform a PCR.

## Conclusions

Gilts were more frequently *M. hyopneumoniae* infected than sows and the highest proportion of infected breeding animals was found in the first half of gestation. Therefore, good acclimation practices for gilts, like proper vaccination and a sufficiently long quarantine period, remain necessary to lower the risk of transmission of *M. hyopneumoniae* in the breeding population and to control the prevalence of *M. hyopneumoniae* in their offspring. Furthermore, vaccination of gilts against *M. hyopneumoniae* is commonly practiced resulting in a high proportion of animals with *M. hyopneumoniae*-specific antibodies. Therefore, methods relying on direct detection of (parts of) the pathogen, such as PCR analysis on TBS are recommended to investigate the *M. hyopneumoniae* infection status of breeding herds.

## Supplementary Information

**Additional file 1.** Detailed results from the statistical models used to analyse the infection status (PCR) data.

**Additional file 2.** Detailed results from the statistical models used to analyse the serology (ELISA) data.

## Data Availability

The datasets used and/or analysed during the current study are available from the corresponding author on reasonable request.
