# Supplementary material for: Influence of parity and reproductive stage on the prevalence of Mycoplasma hyopneumoniae in breeding animals in belgian farrow-to-finish pig herds
Source: Porcine Health Manag. 2022 Jun 9;8:26. doi: 10.1186/s40813-022-00267-w (PMC9178894; doi:10.1186/s40813-022-00267-w)
Supplement: Supplementary file 1 — Additional file 1. Detailed results from the statistical models used to analyse the infection status (PCR) data. [file 40813_2022_267_MOESM1_ESM.pdf]

Additional file 1: Detailed results from the statistical models used to analyse the infection status (PCR) data.

Generalized linear mixed model with farm included as random factor, time point as fixed factor and parity used as binary (gilt or sow).

Fixed Effects[a]

Source	F	df1	df2	Sig.
Corrected Model	13,961	4	795	<0,001
Timepoint	10,808	3	795	<0,001
parity1	26,486	1	795	<0,001

Probability distribution: Binomial

Link function: Logit , a. Target: PCR pos

Estimates

			95% Confidence Interval	
parity1	Mean	Std. Error	Lower	Upper
parity >1	,058	,029	,021	,149
parity 1	,162	,070	,065	,348

Estimates

			95% Confidence Interval	
Time point	Mean	Std. Error	Lower	Upper
30-40 days of gestation	,217	,090	,090	,439
80-90 days of gestation	,107	,052	,040	,259
3-5 days after farrowing	,048	,026	,016	,133
around weaning	,078	,040	,028	,199

Pairwise contrasts

Time point Pairwise Contrasts	Contrast Estimate	Std. Error	t	Adj. Sig.	95% Confidence Interval	
					Lower	Upper
30-40 days of gestation - 80-90 days of gestation	,110	,052	2,108	,035	,008	,212
30-40 days of gestation - 3-5 days after farrowing	,169	,070	2,420	,016	,032	,307
30-40 days of gestation - around weaning	,140	,060	2,329	,020	,022	,257
80-90 days of gestation - 30-40 days of gestation	-,110	,052	-2,108	,035	-,212	-,008
80-90 days of gestation - 3-5 days after farrowing	,060	,034	1,734	,083	-,008	,127
80-90 days of gestation - around weaning	,030	,029	1,045	,296	-,026	,086
3-5 days after farrowing - 30-40 days of gestation	-,169	,070	-2,420	,016	-,307	-,032
3-5 days after farrowing - 80-90 days of gestation	-,060	,034	-1,734	,083	-,127	,008
3-5 days after farrowing - around weaning	-,030	,024	-1,237	,217	-,077	,017
around weaning - 30-40 days of gestation	-,140	,060	-2,329	,020	-,257	-,022
around weaning - 80-90 days of gestation	-,030	,029	-1,045	,296	-,086	,026
around weaning - 3-5 days after farrowing	,030	,024	1,237	,217	-,017	,077

Pairwise comparison between the different time points only for the gilts

Estimates

Time point	Mean	Std. Error	95% Confidence Interval	
			Lower	Upper
30-40 days of gestation	,343	,155	,119	,668
80-90 days of gestation	,148	,090	,041	,413
3-5 days after farrowing	,038	,028	,009	,154
around weaning	,094	,061	,025	,298

Pairwise contrasts

Time point Pairwise Contrasts	Contrast Estimate	Std. Error	t	Adj. Sig.	95% Confidence Interval	
					Lower	Upper
30-40 days of gestation - 80-90 days of gestation	,195	,094	2,074	,039	,010	,379
30-40 days of gestation - 3-5 days after farrowing	,305	,134	2,274	,024	,041	,568
30-40 days of gestation - around weaning	,249	,110	2,269	,024	,033	,466
80-90 days of gestation - 30-40 days of gestation	-,195	,094	-2,074	,039	-,379	-,010
80-90 days of gestation - 3-5 days after farrowing	,110	,070	1,575	,116	-,027	,247
80-90 days of gestation - around weaning	,055	,052	1,051	,294	-,048	,157
3-5 days after farrowing - 30-40 days of gestation	-,305	,134	-2,274	,024	-,568	-,041
3-5 days after farrowing - 80-90 days of gestation	-,110	,070	-1,575	,116	-,247	,027

3-5 days after farrowing - around weaning	-,055	,043	-1,288	,199	-,140	,029
around weaning - 30-40 days of gestation	-,249	,110	-2,269	,024	-,466	-,033
around weaning - 80-90 days of gestation	-,055	,052	-1,051	,294	-,157	,048
around weaning - 3-5 days after farrowing	,055	,043	1,288	,199	-,029	,140

Pairwise comparison between the different time points only for the sows.

Estimates

Time point	Mean	Std. Error	95% Confidence Interval	
			Lower	Upper
30-40 days of gestation	,130	,054	,055	,278
80-90 days of gestation	,076	,036	,030	,183
3-5 days after farrowing	,057	,028	,021	,145
around weaning	,066	,032	,025	,165

Pairwise contrasts

Time point Pairwise Contrasts	Contrast Estimate	Std. Error	t	df	Adj. Sig.	95% Confidence Interval	
						Lower	Upper
30-40 days of gestation - 80-90 days of gestation	,054	,043	1,251	452	,211	-,031	,138
30-40 days of gestation - 3-5 days after farrowing	,073	,044	1,664	452	,097	-,013	,158
30-40 days of gestation - around weaning	,064	,044	1,468	452	,143	-,022	,149
80-90 days of gestation - 30-40 days of gestation	-,054	,043	-1,251	452	,211	-,138	,031
80-90 days of gestation - 3-5 days after farrowing	,019	,030	,638	452	,524	-,040	,078
80-90 days of gestation - around weaning	,010	,031	,331	452	,741	-,051	,072

3-5 days after farrowing - 30-40 days of gestation	-,073	,044	-1,664	452	,097	-,158	,013
3-5 days after farrowing - 80-90 days of gestation	-,019	,030	-,638	452	,524	-,078	,040
3-5 days after farrowing - around weaning	-,009	,028	-,306	452	,760	-,065	,047
around weaning - 30-40 days of gestation	-,064	,044	-1,468	452	,143	-,149	,022
around weaning - 80-90 days of gestation	-,010	,031	-,331	452	,741	-,072	,051
around weaning - 3-5 days after farrowing	,009	,028	,306	452	,760	-,047	,065

Generalized linear mixed model with farm included as random factor, time point as fixed factor and parity used as categorical (gilt, 2-4th parity, >4th parity).

Fixed Effects[a]

Source	F	df1	df2	Sig.
Corrected Model	11,629	5	794	,000
Timepoint	11,310	3	794	,000
paritygroups	14,074	2	794	,000

Probability distribution: Binomial

Link function: Logit

a. Target: PCR pos

Estimates

parity groups	Mean	Std. Error	95% Confidence Interval Lower	95% Confidence Interval Upper
parity 1	,165	,070	,068	,348
parities 2-4	,071	,035	,027	,177
parity >4	,030	,019	,009	,099

Pairwise contrasts

parity groups Pairwise Contrasts	Contrast Estimate	Std. Error	t	df	Adj. Sig.	95% Confidence Interval	
						Lower	Upper
parity 1 - parities 2-4	,094	,042	2,261	794	,024	,012	,175
parity 1 - parity >4	,135	,057	2,347	794	,019	,022	,248
parities 2-4 - parity 1	-,094	,042	-2,261	794	,024	-,175	-,012
parities 2-4 - parity >4	,041	,025	1,610	794	,108	-,009	,091
parity >4 - parity 1	-,135	,057	-2,347	794	,019	-,248	-,022
parity >4 - parities 2-4	-,041	,025	-1,610	794	,108	-,091	,009

The sequential Sidak adjusted significance level is 0.05.

Confidence interval bounds are approximate.
