# Supplementary material for: Influence of parity and reproductive stage on the prevalence of Mycoplasma hyopneumoniae in breeding animals in belgian farrow-to-finish pig herds
Source: Porcine Health Manag. 2022 Jun 9;8:26. doi: 10.1186/s40813-022-00267-w (PMC9178894; doi:10.1186/s40813-022-00267-w)
Supplement: Supplementary file 2 — Additional file 2. Detailed results from the statistical models used to analyse the serology (ELISA) data. [file 40813_2022_267_MOESM2_ESM.pdf]

Additional file 2: Detailed results from the statistical models used to analyse the serology (ELISA) data.

Generalized linear mixed model with farm included as random factor, time point as fixed factor and parity used as binary (gilt or sow).

Fixed Effects[a]

Source	F	df1	df2	Sig.
Corrected Model	5,861	4	750	,000
parity1	9,699	1	750	,002
Timepoint	4,360	3	750	,005

Probability distribution: Binomial

Link function: Logit

a. Target: ELISA pos

Estimates

			95% Confidence Interval	
parity1	Mean	Std. Error	Lower	Upper
parity >1	,861	,053	,722	,936
parity 1	,931	,030	,844	,971

Pairwise Contrasts

						95% Confidence Interval	
parity1 Pairwise Contrasts	Contrast Estimate	Std. Error	t	df	Adj. Sig.	Lower	Upper
parity >1 - parity 1	-,071	,032	-2,219	750	,027	-,133	-,008
parity 1 - parity >1	,071	,032	2,219	750	,027	,008	,133

The sequential Sidak adjusted significance level is .05.

Confidence interval bounds are approximate.

Estimates

Time point	Mean	Std. Error	95% Confidence Interval	
			Lower	Upper
30-40 days of gestation	,898	,044	,774	,958
80-90 days of gestation	,941	,028	,856	,977
3-5 days after farrowing	,832	,065	,665	,925
around weaning	,911	,039	,799	,963

No statistical significant difference for each pairwise comparison.

Generalized linear mixed model with farm included as random factor, time point as fixed factor and parity used as categorical (gilt, 2-4th parity, >4th parity).

Fixed Effects[a]

Source	F	df1	df2	Sig.
Corrected Model	7,497	5	749	,000
Timepoint	5,462	3	749	,001
paritygroups	13,014	2	749	,000

Probability distribution: Binomial

Link function: Logit

a. Target: ELISA pos

Estimates

parity groups	Mean	Std. Error	95% Confidence Interval	
			Lower	Upper
parity 1	,930	,029	,846	,970
parities 2-4	,910	,037	,807	,961
parity >4	,758	,083	,563	,884

Pairwise Contrasts

parity groups Pairwise Contrasts	Contrast Estimate	Std. Error	t	df	Adj. Sig.	95% Confidence Interval	
						Lower	Upper
parity 1 - parities 2-4	,020	,023	,882	749	,378	-,024	,064
parity 1 - parity >4	,172	,063	2,734	749	,019	,021	,322
parities 2-4 - parity 1	-,020	,023	-,882	749	,378	-,064	,024
parities 2-4 - parity >4	,152	,059	2,572	749	,020	,020	,284
parity >4 - parity 1	-,172	,063	-2,734	749	,019	-,322	-,021
parity >4 - parities 2-4	-,152	,059	-2,572	749	,020	-,284	-,020

The sequential Sidak adjusted significance level is .05.

Confidence interval bounds are approximate.
